# Editorial: Clinical practices for co-occurring psychiatric and addictive disorders

**DOI:** 10.3389/fpsyt.2022.1097424

**Published:** 2022-12-20

**Authors:** Alessio Picello, Giuseppe Ducci

**Affiliations:** Azienda Sanitaria Locale Roma 1, Rome, Italy

**Keywords:** dual disorders, addiction, psychiatric comorbidities, substance use disorder, internet use disorder

Dual disorder describes the co-occurrence of addictive and psychiatric disorders, which are increasingly observed when assessing patients with serious mental illnesses. Treatment of patients diagnosed with a coexisting psychiatric and psychoactive substance use disorder remains an important clinical challenge in mental health since this condition leads to worse outcomes and treatment difficulties. There is a lack of knowledge about the elements that need to be targeted so that treatment is effective at softening the impact of an ongoing illness and improving, as much as possible, the ability to function.

This Research Topic features articles that show the effects of addictive disorder on a number of elements, such as quality of life, circadian functioning, anxiety, and depression. In a naturalistic open-label follow-up study on depression in the South Ostrobothnia hospital district of Finland, Luoto et al. found that psychosocial treatment interventions are less effective in patients with depressive symptoms and alcohol use disorder than in patients who only have depressive symptoms, suggesting that comorbid alcohol use problems, as well as anxiety disorders, should be taken into account in the early phase of treatment since both are detrimental to the quality of life among patients with clinical depression.

Dieris-Hirche et al. conducted a cross-sectional study in a German male outpatient clinic and found that people with internet use disorder generally show a reduction in quality of life as measured with WHOQOL, but patients with comorbid mental disorders had a 20.9% lower score than internet use disorder patients without any comorbid mental disorders, suggesting that strategies to reduce depressive symptoms have to be considered in the treatment of internet use disorder patients. Hashemzadeh et al. analyzed the characteristics of the circadian functioning in Iranian patients with substance use disorder with and without major depressive disorder: the comorbid patients had worse sleep quality, and Pittsburgh Sleep Quality Index scores were negatively related to the age of substance use onset and positively related to the severity of depression, suggesting the importance of a precise assessment and early treatment of sleep disturbances.

Huang et al. conducted a cross-sectional study in which over 10,000 9–18-year-old Chinese students completed an online questionnaire. Analysis of the results from this questionnaire validated the hypotheses that anxiety and internet gaming disorder are significantly related: a higher level of anxiety correlates with a higher risk of internet disorder, and frequent and excessive gaming is a temporary coping strategy for people experiencing negative mental states.

The articles in this Research Topic support and extend previous findings indicating that dual diagnosis is associated with worse clinical characteristics, more sleep problems, and poorer quality of life. These results underline the importance of a precise assessment of these measurements in future studies conducted on substance use and other addictive disorders in patients with or without severe mental illness comorbidity.

In conclusion, we collected studies from four different countries showing that dual diagnosis is still considered to be an unsolved problem from a diagnostic and therapeutic point of view. Further studies are needed to address the challenges associated with the treatment of co-occurring disorders.

## Author contributions

AP and GD have contributed to the writing of this editorial. Both authors contributed to the article and approved the submitted version.

